# Supplementary material for: Human-socialized dogs and wolves show similar behavioural reactions to human fear odour
Source: Anim Cogn. 2026 Jul 13;29(1):70. doi: 10.1007/s10071-026-02083-z (PMC13654890; doi:10.1007/s10071-026-02083-z)
Supplement: Supplementary file 1 [file 10071_2026_2083_MOESM1_ESM.docx]

Supplementary Material

**Title:** Human-socialized dogs and wolves show similar behavioural reactions to human fear odour

**Authors:** Svenja Capitain^1*^, Sarah Marshall-Pescini^1^ & Friederike Range^1^

^1^ Domestication Lab, Konrad Lorenz Institute of Ethology, University of Veterinary Medicine Vienna, Austria

* svenja.capitain@vetmeduni.ac.at, friederike.range@vetmeduni.ac.at

**Published in:** Animal Cognition, 2026

Content

[1	Sample collection – movie scenes	1](#_Toc218665528)

[2	Analysis of anxiety scores	1](#_Toc218665529)

[3	Sample processing	2](#_Toc218665530)

[4	Training procedure	3](#_Toc218665531)

[5	Interrater reliability	3](#_Toc218665532)

[6	Detailed model output	3](#_Toc218665533)

[6.1	Interaction Smell * Condition – just wolves	3](#_Toc218665534)

[5.1.1 Choice	3](#_Toc218665535)

[5.1.2 Side preference	4](#_Toc218665536)

[5.1.3 Proximity to targets – test phase	5](#_Toc218665537)

[5.1.4 Proximity to target – Guided phase	5](#_Toc218665538)

[5.1.5 Sniffing the targets	6](#_Toc218665539)

[5.1.6 Engaging	6](#_Toc218665540)

[5.1.7 Looking at target – Test phase	6](#_Toc218665541)

[5.1.8 Looking at targets – Guided phase	7](#_Toc218665542)

[5.1.9 Number of commands	7](#_Toc218665543)

[5.1.10 Latency	8](#_Toc218665544)

[6.2	Interaction Smell * Condition – just dogs	8](#_Toc218665545)

[5.2.1 Choice	8](#_Toc218665546)

[5.2.2 Side preference	9](#_Toc218665547)

[5.2.3 Proximity to target – Test phase	9](#_Toc218665548)

[5.2.4 Proximity to target – Guided phase	10](#_Toc218665549)

[5.2.5 Sniffing target	10](#_Toc218665550)

[5.2.6 Engaging with target	10](#_Toc218665551)

[5.2.7 Looking at target – Test phase	11](#_Toc218665552)

[5.2.8 Looking at targets – Guided phase	11](#_Toc218665553)

[5.2.9 Number of commands	12](#_Toc218665554)

[5.2.10 Latency	12](#_Toc218665555)

[6.3	Target-directed: Interaction Species * Condition	13](#_Toc218665556)

[5.3.1 Choice	13](#_Toc218665557)

[5.3.2 Proximity to targets – Test phase	13](#_Toc218665558)

[5.3.3 Proximity to the target – Giuded phase	14](#_Toc218665559)

[5.3.4 Sniffing	14](#_Toc218665560)

[5.3.5 Engaging with targets	15](#_Toc218665561)

[5.3.6 Looking at the targets – Test phase	15](#_Toc218665562)

[5.3.7 Looking at the targets – Guided phase	16](#_Toc218665563)

[5.3.8 Number of commands	16](#_Toc218665564)

[5.3.9 Latency	17](#_Toc218665565)

[6.4	Non-target directed: Interaction Species * Condition	17](#_Toc218665566)

[5.4.1 Looking at experimenter	17](#_Toc218665567)

[5.4.2 Proximity to experimenter	18](#_Toc218665568)

[5.4.3 High tail	18](#_Toc218665569)

[5.4.4 Low tail	19](#_Toc218665570)

[5.4.5 Not accomplished	19](#_Toc218665571)

[5.4.6 Total trials	20](#_Toc218665572)

[5.4.7 Termination	20](#_Toc218665573)

[5.4.8 Displacement – Test phase	21](#_Toc218665574)

[5.4.9 Nose licks – Guided phase	21](#_Toc218665575)

# 1 Sample collection – movie scenes

Neutral movie scenes were taken from and validated in de Groot, J. H., Smeets, M. A., & Semin, G. R. (2015). *Rapid stress system drives chemical transfer of fear from sender to receiver*. PLOS ONE, 10(2), e0118211. <https://doi.org/10.1371/journal.pone.0118211>.

- We selected two clips for the neutral condition: Clip 23t (8 min): a first-person view of a car and boat traveling through the Dutch countryside. Clip 27t (13 min, 26 s): an excerpt from the TV show “Rail Away”, displayed a train traveling through the Alps.

Fear movie scenes were validated in de Groot, J. H., Kirk, P. A., & Gottfried, J. A. (2020). *Encoding fear intensity in human sweat*. Philosophical Transactions of the Royal Society B, 375(1800), 20190271. <https://doi.org/10.1098/rstb.2019.0271>. They were made available online by de Groot, J. H., Kirk, P. A., & Gottfried, J. A. (2021). *Titrating the smell of fear: Initial evidence for dose-invariant behavioral, physiological, and neural responses*. Psychological Science, 32(4), 558-572. <https://doi.org/10.1177/0956797620970548>.

- We selected four clips for the fear condition: Clip f12 (3 min, 22 s): “The passenger”. Clip f8 (4 min, 35 s): “Nighty Night, Nancy”. Clip f4 (11 min, 25 s): “Vicious”. Clip f9 (3 min, 28 s): “Mr Creak”.

# 2 Analysis of anxiety scores

Odour donators filled out the Spielberger’s State-Trait Anxiety Inventory (Spielberg et al., 1971, German translation from Grimm, 2009), a 10-item questionnaire assessing the current relaxation and nervousness-level across 7-point Likert scales, right before and after watching each movie. A paired t-test was used for each participant and condition to assess whether the scores before and after watching a movie were significantly different. All participants were significantly more stressed after watching the fear movie (p≤0.01), while this was not the case for anyone after watching the neutral movie (p>0.05).

Spielberger CD, Gonzalez-Reigosa F, Martinez-Urrutia A, Natalicio LF, Natalicio DS. The state-trait anxiety inventory. Revista Interamericana de Psicologia/Interamerican journal of psychology. 1971;5(3 & 4).

Grimm J. State-Trait-Anxiety Inventory according to Spielberger. German long and short version.-Methods Forum of the University of Vienna. MF-Working Paper 2009/02; 2009.

# 3 Sample processing

Each odour donor donated eight samples (one pad per axillary, each cut into four pieces) per movie – two times neutral odour, followed by one time fear. Each piece was placed in a sample tube which was labelled with the condition (Neutral 1/ Neutral2/Fear). At the freezer, the sample was moved into a different sampling tube that held a piece from a different person in the same condition. The tube was labelled with a number plus a star or a 0. The number and donator identity were not connected on the sampling sheet, allowing for full anonymity. A sample from the same two people but the other sampling (first vs second neutral movie or first neutral vs fear movie) was placed in a second tube with the same number and a 0 or star respectively. Which numbers were combined and which number and star/0 combination connected to which smell and condition was noted in a master sheet. Which smell (Neutral 1/ Neutral2/Fear) was inside the tube was noted on the tube as well, but covered with a sticker to ensure that the experimenter was blind to the condition and smell. The smell collection and processing commenced four weeks before the start of the experiment.

On the testing sheet, the tube numbers – without the information of their content or condition – were connected to the trial sequence: in what sequence the targets were exchanged between the left and right side during the six test trials, and whether the animal was led to the left or right first during the guided phase. The animals were randomly allocated to a sample pair number.

On the test day, the experimenter took the sample tube pair with the number indicated in the testing sheet out of the freezer for the respective animal. The tube with the star was always placed in the right target first, and the trial sequence indicated in the sheet was followed. The experimenter was hence completely blind which group the animal belonged to and which target held which smell. After the test, to counteract problems due to possible mistakes during the target switching, the stickers covering the smell and condition was taken off facing the camera – thereby still leaving the experimenter blind – with the info which side each tube was in in the last trial. This info was saved separately from the test video for the coding. This way, the experimenter continued to be blind to smells and conditions during coding. But, after the coding, it could be verified that the smells had ended up in the correct sequence as they had been indicated on the sampling sheet. This procedure revealed that no mistakes had been made.

# 4 Training procedure

If the animal did not accomplish this threshold within six trials in the motivation trials, a training session commenced instead. Therein, the handler asked for the command from within 50 cm from the target, increasing the distance by 50 cm whenever the command was accomplished twice. The training session was finished once the animal accomplished the command twice from 3 metre distance or after 20 minutes, whichever came first. The test session was reattempted on a different day.

# 5 Interrater reliability

| **BEHAVIOUR** | | **INTERRATER (ICC)** |
| --- | --- | --- |
| **Guided phase** | | |
|  | Looking at target (D) | 0.8 |
|  | Sniffing target (D) | 0.8 |
|  | Displacement/Self-directed behaviours (F) | 0.82 |
|  | Proximity to target (D) | 0.85 |
| **Test phase** | | |
|  | Proximity to target (D) | 0.96 |
|  | Engaging with target (D) | 0.83 |
|  | Looking at target (D) | 0.96 |
|  | Number of commands (F) | 0.95 |
|  | Command accomplished (B) | 1 |
|  | Latency to accomplish command | 0.98 |
|  | Not accomplished (B) | 1 |
|  | Termination (B) | 1 |
|  | Tail wagging high (D) | 0.98 |
|  | Tail wagging low (D) | 0.94 |
|  | Looking at handler (D) | 0.83 |
|  | Proximity to handler (D) | 0.86 |
|  | Displacement behaviours (F) | 0.80 |
| **Sum** | | **0.90** |

Supplement Table A: Inter-class correlation coefficients (ICC) for each behavior. Twenty percent of all videos (n=8 videos) were re-coded by a second, hypothesis-blind coder who was likewise blind to condition and smell location.

# 6 Detailed model output

Detailed model outputs for each model referenced in the result section. Includes model formula, full-null model comparison (using a likelihood ratio test, with the null model retaining control predictors and random effects), model summary, Tukey-adjusted pairwise comparisons (emmeans) where applicable, and 95% Confidence Intervals (Parametric bootstrapping).

## 6.1 Interaction Smell * Condition – just wolves

### 6.1.1 Choice

Formula: full.W.choice.wc <- glmer(Command.accomplished ~ Condition.y*Smell + Age.z + Sex + Trial.z + (1+Condition.y.F*Smell.Smell2 + Trial.z|AnimalID), family=binomial, data=W.choice)

Full-null model comparison:

|  | npar | AIC | BIC | logLik | Chisq | Df | Pr(>Chisq) |
| --- | --- | --- | --- | --- | --- | --- | --- |
| null.W.choice | 9 | 186.54 | 213.4 | -84.27 |  |  |  |
| full.W.choice | 12 | 190.09 | 225.9 | -83.05 | 2.45 | 3 | 0.48 |

Summary:

|  | Estimate | Std. Error | z-value | p-value | upperCI | lowerCI |  |
| --- | --- | --- | --- | --- | --- | --- | --- |
| (Intercept) | -1.83 | 0.70 | -2.63 | 0.01 | -4.04 | -0.79 |  |
| Condition.yF | 1.05 | 0.66 | 1.60 | 0.11 | -0.11 | 2.84 |  |
| SmellSmell2 | 0.76 | 0.74 | 1.02 | 0.31 | -0.20 | 2.76 |  |
| Age.z | 0.34 | 0.37 | 0.92 | 0.36 | -0.25 | 1.14 |  |
| SexM | 0.17 | 0.71 | 0.25 | 0.81 | -1.16 | 2.03 |  |
| Trial.z | -0.16 | 0.34 | -0.47 | 0.64 | -0.71 | 0.23 |  |
| Condition.yF:SmellSmell2 | -1.30 | 0.97 | -1.34 | 0.18 | -3.83 | 0.39 |  |

### 6.1.2 Side preference

Formula: full.W.side.wc=glmer(Command.accomplished	~ Side.Behaviour*Condition.y + Age.z +	(1+Side.Behaviour.R+Condition.y.F+Trial.z|AnimalID), family=binomial, data=W.side)

Full-null model:

|  | npar | AIC | BIC | logLik | Chisq | Df | Pr(>Chisq) |
| --- | --- | --- | --- | --- | --- | --- | --- |
| null.W.side | 8 | 172.95 | 196.82 | -78.47 |  |  |  |
| full.W.side | 11 | 160.98 | 193.8 | -69.48 | 17.97 | 3 | <0.001 |

Summary:

|  | Estimate | Std. Error | z-value | p-value | upperCI | lowerCI |
| --- | --- | --- | --- | --- | --- | --- |
| (Intercept) | -1.09 | 0.71 | -1.52 | 0.13 | 0.71 | 2.20 |
| SmellSmell2 | -0.79 | 0.64 | -1.24 | 0.22 | -0.81 | 0.37 |
| Condition.yF | 1.26 | 0.58 | 2.19 | 0.03 | -1.18 | -0.06 |
| Age.z | 0.46 | 0.48 | 0.96 | 0.34 | -0.78 | 0.22 |
| SexM | 0.11 | 0.93 | 0.12 | 0.90 | -1.47 | 0.25 |
| Trial.z | -0.17 | 0.43 | -0.39 | 0.69 | -0.33 | 0.01 |
| SmellSmell2:Condition.yF | -2.55 | 0.99 | -2.58 | 0.01 | -0.39 | 1.07 |

Post-hoc:

| contrast | estimate | SE | df | z.ratio | p.value |
| --- | --- | --- | --- | --- | --- |
| LC-RC | 0.79 | 0.64 | Inf | 1.24 | 0.60 |
| LC-LF | -1.26 | 0.58 | Inf | -2.19 | 0.13 |
| LC-RF | 2.08 | 0.79 | Inf | 2.63 | 0.04 |
| RC-LF | -2.05 | 0.64 | Inf | -3.22 | 0.01 |
| RC-RF | 1.29 | 0.82 | Inf | 1.57 | 0.39 |
| LF-RF | 3.34 | 0.77 | Inf | 4.33 | 0.00 |

### 6.1.3 Proximity to targets – test phase

Formula: red.W.proximityT.nonProp <-lmer(Proximity.to.target.T.nonProp_log ~ Smell + Condition.y + Age.z + Sex + Trial.z + (1+Smell.Smell2*Condition.y.F+Trial.z|AnimalID), data = W.proximityT.nonProp)

Full-null model:

|  | npar | AIC | BIC | logLik | Chisq | Df | Pr(>Chisq) |
| --- | --- | --- | --- | --- | --- | --- | --- |
| null.W.proximityT.nonProp | 10 | 103.71 | 122.21 | -41.85 |  |  |  |
| full.W.proximityT.nonProp | 13 | 101.42 | 125.47 | -37.70 | 8.2875 | 3 | 0.04 |

Summary:

|  | Estimate | Std. Error | z-value | upperCI | lowerCI |
| --- | --- | --- | --- | --- | --- |
| (Intercept) | 1.24 | 0.36 | 3.47 | 0.53 | 1.92 |
| Condition.yF | -0.42 | 0.18 | -2.34 | -0.78 | -0.05 |
| SmellSmell2 | 0.01 | 0.17 | 0.09 | -0.29 | 0.38 |
| Age.z | -0.28 | 0.25 | -1.10 | -0.76 | 0.24 |
| SexM | -0.59 | 0.48 | -1.23 | -1.52 | 0.32 |
| Trial.z | -0.17 | 0.08 | -2.12 | -0.33 | -0.01 |

Posthoc:

| contrast | estimate | SE | df | t.ratio | p.value |
| --- | --- | --- | --- | --- | --- |
| C - F | 0.42 | 0.24 | 3.10 | 1.80 | 0.17 |

### 6.1.4 Proximity to target – Guided phase

Formula: full.W.proximityG.nonProp <- lmer(Proximity.to.target.G.nonProp_log	~ Smell	* Condition.y + Age.z + Sex + Trial.z + (1+Smell.Smell2*Condition.y.F+Trial.z|AnimalID), data = W.proximityG.nonProp)

Full-null model:

|  | npar | AIC | BIC | logLik | Chisq | Df | Pr(>Chisq) |
| --- | --- | --- | --- | --- | --- | --- | --- |
| full.W.proximityG.nonProp | 13 | 222.52 | 261.31 | -98.25 |  |  |  |
| null.W.proximityG.nonProp | 20 | 231.87 | 291.54 | -95.93 | 4.64 | 7 | 0.70 |

Summary:

|  | Estimate | Std. Error | z-value | upperCI | lowerCI |
| --- | --- | --- | --- | --- | --- |
| (Intercept) | 1.60 | 0.12 | 13.69 | 1.37 | 1.84 |
| SmellSmell2 | 0.12 | 0.16 | 0.73 | -0.203 | 0.43 |
| Condition.yF | 0.00 | 0.17 | -0.03 | -0.339 | 0.335 |
| Age.z | -0.16 | 0.04 | -4.07 | -0.241 | -0.076 |
| SexM | -0.01 | 0.08 | -0.15 | -0.187 | 0.149 |
| Trial.z | 0.05 | 0.06 | 0.77 | -0.075 | 0.165 |
| SmellSmell2:Condition.yF | -0.11 | 0.25 | -0.44 | -0.594 | 0.39 |

### 6.1.5 Sniffing the targets

Formula: full.W.sniffingTP <- glmmTMB(Sniffing.target.G ~ Smell	* Condition.y + Age.z + Sex + Trial.z +	(1+Smell.Smell2*Condition.y.F+Trial.z|AnimalID), family	= beta_family, data = W.sniffingT)

Full-null model:

|  | npar | AIC | BIC | logLik | Chisq | Df | Pr(>Chisq) |
| --- | --- | --- | --- | --- | --- | --- | --- |
| null.SniffTP | 8 | -1170.3 | -1137.8 | 593.15 |  |  |  |
| full.Both.sniffingTP | 11 | -1169.5 | -1124.8 | 595.74 | 5.17 | 3 | 0.16 |

Summary:

|  | Estimate | Std. Error | z-value | p-value | upperCI | lowerCI |
| --- | --- | --- | --- | --- | --- | --- |
| (Intercept) | -1.89 | 0.26 | -7.21 | 0.00 | -2.47 | -1.38 |
| SmellSmell2 | -0.03 | 0.26 | -0.12 | 0.90 | -0.51 | 0.46 |
| Condition.yF | -0.05 | 0.24 | -0.19 | 0.85 | -0.54 | 0.43 |
| Age.z | 0.05 | 0.16 | 0.30 | 0.77 | -0.24 | 0.36 |
| SexM | 0.35 | 0.32 | 1.10 | 0.27 | -0.28 | 0.95 |
| Trial.z | -0.28 | 0.09 | -3.06 | 0.00 | -0.47 | -0.08 |
| SmellSmell2:Condition.yF | -0.26 | 0.34 | -0.77 | 0.44 | -0.95 | 0.38 |

### 6.1.6 Engaging

Formula: full.W.engagingTP.wc	<- glmmTMB(Engaging.with.target.T ~ Smell * Condition.y + Age.z + Sex + Trial.z + (1+Smell.Smell2*Condition.y.F+Trial.z|AnimalID), family =	beta_family)

Full-null model:

|  | npar | AIC | BIC | logLik | Chisq | Df | Pr(>Chisq) |
| --- | --- | --- | --- | --- | --- | --- | --- |
| null.EngagTP | 8 | -104.44 | -82.01 | 60.22 |  |  |  |
| full.Both.engagingTP | 11 | -101.67 | -70.82 | 61.83 | 3.22 | 3 | 0.36 |

Summary:

|  | Estimate | Std. Error | z-value | p-value | upperCI | lowerCI |
| --- | --- | --- | --- | --- | --- | --- |
| (Intercept) | -0.20 | 0.35 | -0.58 | 0.56 | -0.97 | 0.60 |
| SmellSmell2 | -0.16 | 0.37 | -0.44 | 0.66 | -0.94 | 0.62 |
| Condition.yF | -0.41 | 0.31 | -1.32 | 0.19 | -1.14 | 0.24 |
| Age.z | -0.01 | 0.15 | -0.06 | 0.95 | -0.33 | 0.27 |
| SexM | -0.61 | 0.33 | -1.84 | 0.07 | -1.35 | 0.05 |
| Trial.z | -0.12 | 0.11 | -1.14 | 0.25 | -0.34 | 0.09 |
| SmellSmell2:Condition.yF | 0.05 | 0.47 | 0.11 | 0.92 | -0.93 | 0.99 |

### 6.1.7 Looking at target – Test phase

Formula: red.W.LookingT.nonProp <- lmer(Looking.at.target.T.nonProp_log ~ Smell + Condition.y + Age.z +	Sex + Trial.z + (1+Smell.Smell2*Condition.y.F+Trial.z|AnimalID), data = W.LookingT.nonProp)

Full-null model:

|  | npar | AIC | BIC | logLik | Chisq | Df | Pr(>Chisq) |
| --- | --- | --- | --- | --- | --- | --- | --- |
| null.LookTP | 20 | 138.01 | 179.56 | -49.07 |  |  |  |
| full.W.LookingT.nonProp | 22 | 141.59 | 187.3 | -48.78 | 0.42 | 2 | 0.81 |

Summary:

|  | Estimate | Std. Error | z-value | upperCI | lowerCI |
| --- | --- | --- | --- | --- | --- |
| (Intercept) | 0.60 | 0.22 | 2.69 | 0.08 | 1.14 |
| SmellSmell2 | 0.06 | 0.32 | 0.18 | -0.63 | 0.76 |
| Condition.yF | -0.08 | 0.19 | -0.41 | -0.50 | 0.37 |
| Age.z | 0.11 | 0.09 | 1.24 | -0.14 | 0.36 |
| SexM | -0.09 | 0.18 | -0.51 | -0.58 | 0.35 |
| Trial.z | -0.19 | 0.08 | -2.50 | -0.36 | 0.01 |

### 6.1.8 Looking at targets – Guided phase

Formula: full.W.LookingG.nonProp <- lmer(Looking.at.target.G.nonProp_log ~ Smell * Condition.y + Age.z +	Sex + Trial.z + (1+Smell.Smell2*Condition.y.F+Trial.z||AnimalID),	data = W.LookingG.nonProp)

Full-null model:

|  | npar | AIC | BIC | logLik | Chisq | Df | Pr(>Chisq) |
| --- | --- | --- | --- | --- | --- | --- | --- |
| null.W.LookingG.nonProp | 10 | 316.42 | 344.78 | -148.21 |  |  |  |
| full.W.LookingG.nonProp | 13 | 316.2 | 353.07 | -145.1 | 6.23 | 3 | 0.10 |

Summary:

|  | Estimate | Std. Error | z-value | upperCI | lowerCI |
| --- | --- | --- | --- | --- | --- |
| (Intercept) | 0.26 | 0.24 | 1.09 | -0.18 | 0.75 |
| SmellSmell2 | 0.13 | 0.23 | 0.59 | -0.31 | 0.59 |
| Condition.yF | -0.05 | 0.19 | -0.28 | -0.43 | 0.31 |
| Age.z | 0.02 | 0.15 | 0.11 | -0.29 | 0.31 |
| SexM | 0.44 | 0.31 | 1.43 | -0.22 | 1.01 |
| Trial.z | -0.20 | 0.07 | -2.79 | -0.34 | -0.04 |
| SmellSmell2:Condition.yF | -0.41 | 0.27 | -1.51 | -0.95 | 0.13 |

### 6.1.9 Number of commands

Formula: full.W.commands <- glmer(Command.Smell ~ Smell * Condition.y + Age.z + Sex	+ Trial.z	+	(1+Smell.Smell2*Condition.y.F+Trial.z||AnimalID), family = poisson(), data = W.commands)

Full-null model:

|  | npar | AIC | BIC | logLik | Chisq | Df | Pr(>Chisq) |
| --- | --- | --- | --- | --- | --- | --- | --- |
| null.W.commands | 9 | 147.34 | 162.98 | -64.67 |  |  |  |
| full.W.commands | 12 | 149.66 | 170.51 | -62.83 | 3.68 | 3 | 0.30 |

Summary:

|  | Estimate | Std. Error | z-value | upperCI | lowerCI |
| --- | --- | --- | --- | --- | --- |
| (Intercept) | 0.45 | 0.50 | 0.91 | -0.66 | 1.80 |
| SmellSmell2 | 0.04 | 0.47 | 0.08 | -1.04 | 0.95 |
| Condition.yF | -0.38 | 0.45 | -0.83 | -1.74 | 0.71 |
| Age.z | 0.14 | 0.22 | 0.65 | -0.21 | 0.55 |
| SexM | -0.01 | 0.43 | -0.02 | -1.02 | 0.98 |
| Trial.z | 0.26 | 0.25 | 1.04 | -0.19 | 0.62 |
| SmellSmell2:Condition.yF | -0.52 | 0.66 | -0.79 | -2.06 | 0.96 |

### 6.1.10 Latency

Formula: W.full.lat <- lmer(LatencySmell_log ~ Condition.y*Smell + Age.z + Sex +	Trial.z +	(1+Smell.Smell2*Condition.y.F+Trial.z||AnimalID), data	= W.latency)

Full-null model:

|  | npar | AIC | BIC | logLik | Chisq | Df | Pr(>Chisq) |
| --- | --- | --- | --- | --- | --- | --- | --- |
| W.null.lat | 10 | 125.11 | 142.48 | -52.55 |  |  |  |
| W.full.lat | 13 | 127.79 | 150.38 | -50.89 | 3.31 | 3 | 0.35 |

Summary:

|  | Estimate | Std. Error | z-value | upperCI | lowerCI |
| --- | --- | --- | --- | --- | --- |
| (Intercept) | 2.52 | 0.47 | 5.34 | 1.49 | 3.45 |
| Condition.yF | -0.12 | 0.45 | -0.26 | -1.00 | 0.80 |
| SmellSmell2 | 0.24 | 0.48 | 0.49 | -0.70 | 1.29 |
| Age.z | 0.02 | 0.16 | 0.10 | -0.33 | 0.32 |
| SexM | 0.24 | 0.35 | 0.70 | -0.50 | 0.96 |
| Trial.z | 0.26 | 0.24 | 1.11 | -0.22 | 0.72 |
| Condition.yF:SmellSmell2 | -0.50 | 0.59 | -0.86 | -1.68 | 0.75 |

## 6.2 Interaction Smell * Condition – just dogs

### 6.2.1 Choice

Formula: full.D.choice <- glmer(Command.accomplished ~ Condition.y*Smell + Age.z + Sex + Trial.z +	(1+Smell.Smell2*Condition.y.F+Trial.z||AnimalID), family=binomial, data=D.choice)

Full-null model:

|  | npar | AIC | BIC | logLik | Chisq | Df | Pr(>Chisq) |
| --- | --- | --- | --- | --- | --- | --- | --- |
| null.D.choice | 9 | 394.93 | 427.77 | -188.46 |  |  |  |
| full.D.choice | 12 | 399.24 | 443.02 | -187.62 | 1.69 | 3 | 0.64 |

Summary:

|  | Estimate | Std. Error | z-value | p-value | upperCI | lowerCI |
| --- | --- | --- | --- | --- | --- | --- |
| (Intercept) | -0.70 | 0.36 | -1.94 | 0.05 | -1.41 | -0.01 |
| Condition.yF | 0.46 | 0.39 | 1.19 | 0.23 | -0.41 | 1.30 |
| SmellSmell2 | 0.47 | 0.53 | 0.89 | 0.38 | -0.47 | 1.28 |
| Age.z | 0.23 | 0.13 | 1.78 | 0.07 | -0.02 | 0.57 |
| SexM | 0.01 | 0.27 | 0.04 | 0.97 | -0.51 | 0.41 |
| Trial.z | -0.11 | 0.13 | -0.83 | 0.41 | -0.31 | 0.17 |
| Condition.yF:SmellSmell2 | -0.55 | 0.56 | -0.98 | 0.33 | -1.55 | 0.39 |

### 6.2.2 Side preference

Formula: full.D.side = glmer(Command.accomplished ~ Side.Behaviour*Condition.y + Age.z + Sex	+	Trial.z + (1+Side.Behaviour.R*Condition.y.F+Trial.z|AnimalID), family=binomial, data=D.side

Full-null model:

|  | npar | AIC | BIC | logLik | Chisq | Df | Pr(>Chisq) |
| --- | --- | --- | --- | --- | --- | --- | --- |
| null.D.side | 19 | 342.68 | 412.01 | -152.34 |  |  |  |
| full.D.side | 22 | 346.61 | 426.89 | -151.3 | 2.07 | 3 | 0.56 |

Summary:

|  | Estimate | Std. Error | z-value | p-value | upperCI | lowerCI |
| --- | --- | --- | --- | --- | --- | --- |
| (Intercept) | -0.75 | 0.69 | -1.10 | 0.27 | -1.69 | 0.86 |
| Side.BehaviourR | 0.22 | 1.04 | 0.21 | 0.83 | -2.36 | 1.80 |
| Condition.yF | 0.66 | 1.04 | 0.63 | 0.53 | -2.25 | 2.21 |
| Age.z | 0.39 | 0.16 | 2.43 | 0.02 | 0.07 | 0.79 |
| SexM | 0.01 | 0.32 | 0.02 | 0.98 | -0.78 | 0.61 |
| Trial.z | -0.16 | 0.17 | -0.97 | 0.33 | -0.51 | 0.16 |
| Side.BehaviourR:Condition.yF | -0.98 | 1.95 | -0.51 | 0.61 | -4.56 | 3.75 |

### 6.2.3 Proximity to target – Test phase

Formula: full.D.proximityT.nonProp <- lmer(Proximity.to.target.T.nonProp_log ~ Smell * Condition.y +	Age.z + Sex + Trial.z + (1+Smell.Smell2*Condition.y.F+Trial.z||AnimalID),	data =	D.proximityT.nonProp)

Full-null model:

|  | npar | AIC | BIC | logLik | Chisq | Df | Pr(>Chisq) |
| --- | --- | --- | --- | --- | --- | --- | --- |
| full.D.proximityT.nonProp | 13 | 195.99 | 232.86 | -84.99 |  |  |  |
| null.D.proximityT.nonProp | 20 | 210.51 | 267.24 | -85.25 | 0 | 7 | 1 |

Summary:

|  | Estimate | Std. Error | z-value | upperCI | lowerCI |
| --- | --- | --- | --- | --- | --- |
| (Intercept) | 0.69 | 0.11 | 6.33 | 0.48 | 0.91 |
| SmellSmell2 | 0.09 | 0.12 | 0.73 | -0.17 | 0.34 |
| Condition.yF | -0.06 | 0.14 | -0.43 | -0.33 | 0.22 |
| Age.z | -0.08 | 0.06 | -1.41 | -0.19 | 0.03 |
| SexM | -0.04 | 0.11 | -0.32 | -0.27 | 0.18 |
| Trial.z | 0.00 | 0.04 | -0.05 | -0.09 | 0.09 |
| SmellSmell2:Condition.yF | -0.29 | 0.18 | -1.60 | -0.65 | 0.07 |

### 6.2.4 Proximity to target – Guided phase

Formula: full.D.proximityG.nonProp <- lmer(Proximity.to.target.G.nonProp_log ~ Smell *	Condition.y + Age.z	+ Sex + Trial.z +	(1+Smell.Smell2*Condition.y.F+Trial.z|AnimalID), data = D.proximityG.nonProp)

Full-null model:

|  | npar | AIC | BIC | logLik | Chisq | Df | Pr(>Chisq) |
| --- | --- | --- | --- | --- | --- | --- | --- |
| null.D.proximityG.nonProp | 20 | 242.17 | 315.15 | -101.08 |  |  |  |
| full.D.proximityG.nonProp | 23 | 246.6 | 330.53 | -100.3 | 1.56 | 3 | 0.67 |

Summary:

|  | Estimate | Std. Error | z-value | upperCI | lowerCI |
| --- | --- | --- | --- | --- | --- |
| (Intercept) | 1.28 | 0.09 | 14.39 | 1.11 | 1.47 |
| SmellSmell2 | 0.04 | 0.07 | 0.55 | -0.09 | 0.17 |
| Condition.yF | -0.03 | 0.14 | -0.24 | -0.31 | 0.23 |
| Age.z | -0.05 | 0.05 | -1.02 | -0.15 | 0.06 |
| SexM | 0.05 | 0.10 | 0.53 | -0.16 | 0.26 |
| Trial.z | 0.00 | 0.02 | -0.09 | -0.05 | 0.04 |
| SmellSmell2:Condition.yF | -0.09 | 0.08 | -1.05 | -0.26 | 0.07 |

### 6.2.5 Sniffing target

Formula: full.D.sniffingTP <- glmmTMB(Sniffing.target.G	~ Smell	* Condition.y + Age.z + Sex +	Trial.z + (1+Smell.Smell2*Condition.y.F+Trial.z||AnimalID), family = beta_family,	data =	D.sniffingTP)

Full-null model:

|  | npar | AIC | BIC | logLik | Chisq | Df | Pr(>Chisq) |
| --- | --- | --- | --- | --- | --- | --- | --- |
| null.SniffTP | 8 | -1170.3 | -1137.8 | 593.15 |  |  |  |
| full.Both.sniffingTP | 11 | -1169.5 | -1124.8 | 595.74 | 5.17 | 3 | 0.16 |

Summary:

|  | Estimate | Std. Error | z-value | p-value | upperCI | lowerCI |
| --- | --- | --- | --- | --- | --- | --- |
| (Intercept) | -1.92 | 0.17 | -11.26 | <0.001 | -2.29 | -1.63 |
| SmellSmell2 | -0.04 | 0.17 | -0.21 | 0.83 | -0.34 | 0.31 |
| Condition.yF | 0.18 | 0.24 | 0.77 | 0.44 | -0.28 | 0.62 |
| Age.z | 0.13 | 0.07 | 1.81 | 0.07 | -0.01 | 0.27 |
| SexM | 0.18 | 0.14 | 1.26 | 0.21 | -0.08 | 0.45 |
| Trial.z | -0.23 | 0.06 | -3.61 | 0.00 | -0.35 | -0.11 |
| SmellSmell2:Condition.yF | -0.02 | 0.24 | -0.07 | 0.95 | -0.49 | 0.43 |

### 6.2.6 Engaging with target

Formula: full.D.engagingTP <- glmmTMB(Engaging.with.target.T	~ Smell	* Condition.y + Age.z + Sex	+ Trial.z	+ (1+Smell.Smell2*Condition.y.F+Trial.z||AnimalID), family = beta_family, data	=	D.engagingTP)

Full-null model:

|  | npar | AIC | BIC | logLik | Chisq | Df | Pr(>Chisq) |
| --- | --- | --- | --- | --- | --- | --- | --- |
| null.EngagTP | 8 | -104.44 | -82.013 | 60.223 |  |  |  |
| full.Both.engagingTP | 11 | -101.67 | -70.823 | 61.834 | 3.22 | 3 | 0.36 |

Summary:

|  | Estimate | Std. Error | z-value | p-value | upperCI | lowerCI |
| --- | --- | --- | --- | --- | --- | --- |
| (Intercept) | -0.78 | 0.17 | -4.64 | 0.00 | -1.10 | -0.45 |
| SmellSmell2 | 0.41 | 0.20 | 1.98 | 0.05 | -0.02 | 0.81 |
| Condition.yF | -0.09 | 0.24 | -0.39 | 0.69 | -0.54 | 0.32 |
| Age.z | -0.08 | 0.07 | -1.07 | 0.29 | -0.21 | 0.06 |
| SexM | 0.13 | 0.15 | 0.88 | 0.38 | -0.18 | 0.43 |
| Trial.z | -0.20 | 0.10 | -1.88 | 0.06 | -0.41 | 0.00 |
| SmellSmell2:Condition.yF | -0.31 | 0.29 | -1.07 | 0.29 | -0.89 | 0.25 |

### 6.2.7 Looking at target – Test phase

Formula: full.D.LookingT.nonProp <- lmer(Looking.at.target.T.nonProp_log ~ Smell * Condition.y	+	Age.z +	Sex + Trial.z + (1+Smell.Smell2*Condition.y.F+Trial.z||AnimalID),	data =	D.LookingT.nonProp)

Full-null model:

|  | npar | AIC | BIC | logLik | Chisq | Df | Pr(>Chisq) |
| --- | --- | --- | --- | --- | --- | --- | --- |
| null.LookTP | 20 | 138.01 | 179.56 | -49.00 |  |  |  |
| full.W.LookingT.nonProp | 22 | 141.59 | 187.3 | -48.79 | 0.42 | 2 | 0.81 |

Summary:

|  | Estimate | Std. Error | z-value | upperCI | lowerCI |
| --- | --- | --- | --- | --- | --- |
| (Intercept) | 0.56 | 0.17 | 3.26 | 0.22 | 0.88 |
| SmellSmell2 | -0.02 | 0.20 | -0.08 | -0.40 | 0.39 |
| Condition.yF | 0.02 | 0.19 | 0.09 | -0.36 | 0.40 |
| Age.z | -0.01 | 0.10 | -0.09 | -0.20 | 0.17 |
| SexM | -0.14 | 0.19 | -0.76 | -0.48 | 0.25 |
| Trial.z | 0.05 | 0.06 | 0.82 | -0.07 | 0.17 |
| SmellSmell2:Condition.yF | -0.19 | 0.28 | -0.67 | -0.71 | 0.35 |

### 6.2.8 Looking at targets – Guided phase

Formula: full.D.LookingG.nonProp <- lmer(Looking.at.target.G.nonProp_log ~ Smell * Condition.y +	Age.z +	Sex + Trial.z + (1+Smell.Smell2*Condition.y.F+Trial.z|AnimalID), data =	D.LookingG.nonProp)

Full-null model:

|  | npar | AIC | BIC | logLik | Chisq | Df | Pr(>Chisq) |
| --- | --- | --- | --- | --- | --- | --- | --- |
| null.D.LookingG.nonProp | 10 | 602.64 | 638.13 | -291.32 |  |  |  |
| full.D.LookingG.nonProp | 13 | 607.47 | 653.61 | -290.73 | 1.16 | 3 | 0.76 |

Summary:

|  | Estimate | Std. Error | z-value | upperCI | lowerCI |
| --- | --- | --- | --- | --- | --- |
| (Intercept) | 0.30 | 0.14 | 2.10 | 0.02 | 0.59 |
| SmellSmell2 | -0.05 | 0.13 | -0.39 | -0.30 | 0.19 |
| Condition.yF | -0.11 | 0.18 | -0.61 | -0.50 | 0.27 |
| Age.z | 0.05 | 0.08 | 0.58 | -0.10 | 0.20 |
| SexM | -0.05 | 0.16 | -0.32 | -0.37 | 0.27 |
| Trial.z | -0.17 | 0.05 | -3.57 | -0.26 | -0.08 |
| SmellSmell2:Condition.yF | 0.18 | 0.18 | 0.98 | -0.16 | 0.57 |

### 6.2.9 Number of commands

Formula: full.D.commands <- glmer(Command.Smell ~ Smell * Condition.y + Age.z + Sex	+ Trial.z	+	(1+Smell.Smell2*Condition.y.F+Trial.z||AnimalID), family = poisson(), data =	D.commands)

Full-null model:

|  | npar | AIC | BIC | logLik | Chisq | Df | Pr(>Chisq) |
| --- | --- | --- | --- | --- | --- | --- | --- |
| null.D.commands | 9 | 402.14 | 427.07 | -192.07 |  |  |  |
| full.D.commands | 12 | 406.31 | 439.56 | -191.16 | 1.83 | 3 | 0.61 |

Summary:

|  | Estimate | Std. Error | z-value | upperCI | lowerCI |
| --- | --- | --- | --- | --- | --- |
| (Intercept) | 0.85 | 0.16 | 5.49 | 0.61 | 1.07 |
| SmellSmell2 | -0.14 | 0.19 | -0.75 | -0.54 | 0.18 |
| Condition.yF | -0.19 | 0.18 | -1.01 | -0.52 | 0.11 |
| Age.z | -0.11 | 0.08 | -1.35 | -0.24 | 0.00 |
| SexM | 0.13 | 0.15 | 0.86 | -0.12 | 0.33 |
| Trial.z | 0.06 | 0.07 | 0.84 | -0.04 | 0.17 |
| SmellSmell2:Condition.yF | 0.11 | 0.26 | 0.43 | -0.31 | 0.60 |

### 6.2.10 Latency

Formula: D.full.lat <- lmer(LatencySmell_log ~ Condition.y*Smell	+ Age.z	+ Sex +	Trial.z +	(1+Smell.Smell2*Condition.y.F+Trial.z|AnimalID), data =	D.latency)

Full-null model:

|  | npar | AIC | BIC | logLik | Chisq | Df | Pr(>Chisq) |
| --- | --- | --- | --- | --- | --- | --- | --- |
| D.null.lat | 10 | 326.17 | 353.88 | -153.09 |  |  |  |
| D.full.lat | 13 | 331.33 | 367.35 | -152.66 | 0.84 | 3 | 0.84 |

Summary:

|  | Estimate | Std. Error | z-value | upperCI | lowerCI |
| --- | --- | --- | --- | --- | --- |
| (Intercept) | 2.14 | 0.25 | 8.49 | 1.71 | 2.60 |
| Condition.yF | -0.18 | 0.31 | -0.57 | -0.70 | 0.45 |
| SmellSmell2 | 0.13 | 0.25 | 0.53 | -0.34 | 0.61 |
| Age.z | -0.16 | 0.09 | -1.75 | -0.34 | 0.09 |
| SexM | 0.37 | 0.19 | 2.00 | -0.11 | 0.79 |
| Trial.z | 0.14 | 0.10 | 1.35 | -0.08 | 0.31 |
| Condition.yF:SmellSmell2 | -0.08 | 0.37 | -0.22 | -0.80 | 0.60 |

## 6.3 Target-directed: Interaction Species * Condition

### 6.3.1 Choice

Formula: full.Both.choice <- glmer(Command.accomplished ~ Condition.y*Species + Age.z + Sex +	Trial.z + (1+Trial.z+Condition.y.Experimental|AnimalID), family=binomial, data=Both.choice)

Full-null model:

|  | npar | AIC | BIC | logLik | Chisq | Df | Pr(>Chisq) |
| --- | --- | --- | --- | --- | --- | --- | --- |
| null.Both.choice | 7 | 578.64 | 607.09 | -282.32 |  |  |  |
| full.Both.choice | 13 | 579.64 | 632.47 | -276.82 | 10.99 | 6 | 0.08 |

Formula: red.Both.choice <- glmer(Command.accomplished ~ Condition.y + Species + Age.z + Sex +	Trial.z + (1+Trial.z+Condition.y.Experimental||AnimalID), family=binomial, data=Both.choice)

Reduced-null model:

|  | npar | AIC | BIC | logLik | Chisq | Df | Pr(>Chisq) |
| --- | --- | --- | --- | --- | --- | --- | --- |
| null.Both.choice | 7 | 578.64 | 607.09 | -282.32 |  |  |  |
| red.Both.choice | 9 | 571.87 | 608.44 | -276.93 | 10.774 | 2 | <0.005 |

Summary:

|  | Estimate | Std. Error | z-value | p-value | upperCI | lowerCI |
| --- | --- | --- | --- | --- | --- | --- |
| (Intercept) | -0.44 | 0.18 | -2.42 | 0.02 | -0.77 | -0.13 |
| Condition.yExperimental | 0.22 | 0.20 | 1.09 | 0.28 | -0.14 | 0.73 |
| SpeciesWolf | -0.71 | 0.23 | -3.07 | 0.00 | -1.11 | -0.21 |
| Age.z | 0.23 | 0.11 | 2.13 | 0.03 | 0.09 | 0.45 |
| SexM | 0.07 | 0.21 | 0.33 | 0.74 | -0.31 | 0.47 |
| Trial.z | -0.09 | 0.10 | -0.92 | 0.36 | -0.29 | 0.09 |

Posthoc:

| contrast | estimate | SE | df | z.ratio | p.value |
| --- | --- | --- | --- | --- | --- |
| Dog - Wolf | 0.706 | 0.23 | Inf | 3.072 | 0.0021 |

### 6.3.2 Proximity to targets – Test phase

Formula: red.Both.proximityT.nonProp	<- lmer(Proximity.to.target.T.nonProp_log ~ Condition.y	+	Species	+ Age.z	+ Sex +	Trial.z + (1+Trial.z+Condition.y.Experimental||AnimalID), data	=	Both.proximityT.nonProp)

Full-null model:

|  | npar | AIC | BIC | logLik | Chisq | Df | Pr(>Chisq) |
| --- | --- | --- | --- | --- | --- | --- | --- |
| null.Both.proximityT.nonProp | 8 | 291.36 | 316.59 | -137.68 |  |  |  |
| full.Both.proximityT.nonProp | 11 | 281.15 | 315.83 | -129.57 | 16.22 | 3 | <0.002 |

Summary:

|  | Estimate | Std. Error | z-value | p-value | upperCI | lowerCI |
| --- | --- | --- | --- | --- | --- | --- |
| (Intercept) | 0.79 | 0.10 | 7.58 | <0.01 | 0.56 | 1.00 |
| Condition.yExperimental | -0.28 | 0.08 | -3.27 | 0.02 | -0.45 | -0.10 |
| SpeciesWolf | 0.31 | 0.14 | 2.21 | 0.02 | 0.02 | 0.63 |
| Age.z | -0.14 | 0.06 | -2.11 | 0.32 | -0.26 | -0.02 |
| SexM | -0.12 | 0.13 | -0.95 | 0.51 | -0.37 | 0.14 |
| Trial.z | -0.03 | 0.04 | -0.68 | 0.00 | -0.12 | 0.05 |

Posthoc:

| contrast | estimate | SE | df | t.ratio | p.value |
| --- | --- | --- | --- | --- | --- |
| Control - Experimental | 0.28 | 0.09 | 14.90 | 3.21 | 0.01 |
| Dog - Wolf | -0.31 | 0.14 | 15.40 | -2.19 | 0.04 |

### 6.3.3 Proximity to the target – Giuded phase

Formula: full.Both.proximityG.nonProp	<- lmer(Proximity.to.target.G.nonProp_log ~ Species *	Condition.y + Age.z + Sex + Trial.z + (1+Trial.z+Condition.y.Experimental|AnimalID), data	=	Both.proximityG.nonProp)

Full-null model:

|  | npar | AIC | BIC | logLik | Chisq | Df | Pr(>Chisq) |
| --- | --- | --- | --- | --- | --- | --- | --- |
| null.Both.proximityG.nonProp | 11 | 379.51 | 424.21 | -178.75 |  |  |  |
| full.Both.proximityG.nonProp | 14 | 382.68 | 439.58 | -177.34 | 2.82 | 3 | 0.42 |

Summary:

|  | Estimate | Std. Error | z-value | upperCI | lowerCI |
| --- | --- | --- | --- | --- | --- |
| (Intercept) | 1.56 | 0.06 | 24.19 | 1.43 | 1.68 |
| SpeciesWolf | 0.10 | 0.10 | 1.00 | -0.11 | 0.30 |
| Condition.yExperimental | -0.05 | 0.10 | -0.51 | -0.24 | 0.15 |
| Age.z | -0.08 | 0.03 | -2.49 | -0.14 | -0.02 |
| SexM | 0.02 | 0.06 | 0.26 | -0.10 | 0.15 |
| Trial.z | 0.01 | 0.02 | 0.47 | -0.04 | 0.05 |
| SpeciesWolf:Condition.yExperimental | -0.01 | 0.16 | -0.08 | -0.35 | 0.31 |

### 6.3.4 Sniffing

Formula: full.Both.sniffingTP <- glmmTMB(Sniffing.target.G ~ Species * Condition.y + Age.z + Sex	+	Trial.z + (1+Trial.z+Condition.y.Experimental||AnimalID), family	= beta_family, data =	Both.sniffingTP)

Full-null model:

|  | npar | AIC | BIC | logLik | Chisq | Df | Pr(>Chisq) |
| --- | --- | --- | --- | --- | --- | --- | --- |
| null.SniffTP | 8 | -1170.3 | -1137.8 | 593.15 |  |  |  |
| full.Both.sniffingTP | 11 | -1169.5 | -1124.8 | 595.74 | 5.17 | 3 | 0.16 |

Summary:

|  | Estimate | Std. Error | z-value | p-value | upperCI | lowerCI |
| --- | --- | --- | --- | --- | --- | --- |
| (Intercept) | -1.90 | 0.15 | -12.29 | <0.01 | -2.20 | -1.61 |
| SpeciesWolf | -0.14 | 0.23 | -0.63 | 0.53 | -0.60 | 0.33 |
| Condition.yExperimental | 0.17 | 0.18 | 0.99 | 0.32 | -0.18 | 0.52 |
| Age.z | 0.08 | 0.08 | 1.08 | 0.28 | -0.08 | 0.23 |
| SexM | 0.23 | 0.16 | 1.46 | 0.14 | -0.07 | 0.54 |
| Trial.z | -0.25 | 0.05 | -4.64 | 0.00 | -0.35 | -0.15 |
| SpeciesWolf: Condition.yExperimental | -0.35 | 0.30 | -1.19 | 0.24 | -0.92 | 0.18 |

### 6.3.5 Engaging with targets

Formula: full.Both.engagingTP <- glmmTMB(Engaging.with.target.T ~ Species * Condition.y + Age.z	+ Sex +	Trial.z + (1+Trial.z+Condition.y.Experimental||AnimalID), family	= beta_family, data	= Both.engagingTP)

Full-null model:

|  | npar | AIC | BIC | logLik | Chisq | Df | Pr(>Chisq) |
| --- | --- | --- | --- | --- | --- | --- | --- |
| null.EngagTP | 8 | -104.44 | -82.013 | 60.223 |  |  |  |
| full.Both.engagingTP | 11 | -101.67 | -70.823 | 61.834 | 3.22 | 3 | 0.36 |

Summary:

|  | Estimate | Std. Error | z-value | p-value | upperCI | lowerCI |
| --- | --- | --- | --- | --- | --- | --- |
| (Intercept) | -0.50 | 0.13 | -3.99 | <0.001 | -0.76 | -0.27 |
| SpeciesWolf | -0.06 | 0.25 | -0.26 | 0.80 | -0.55 | 0.41 |
| Condition.yExperimental | -0.25 | 0.15 | -1.68 | 0.09 | -0.55 | 0.02 |
| Age.z | -0.03 | 0.08 | -0.35 | 0.72 | -0.17 | 0.12 |
| SexM | -0.11 | 0.16 | -0.69 | 0.49 | -0.43 | 0.19 |
| Trial.z | -0.14 | 0.07 | -1.93 | 0.05 | -0.30 | -0.01 |
| SpeciesWolf: Condition.yExperimental | 0.03 | 0.29 | 0.10 | 0.92 | -0.49 | 0.61 |

### 6.3.6 Looking at the targets – Test phase

Formula: red.Both.LookingTP <-	glmmTMB(Looking.at.target.T ~	Species	+ Condition.y + Age.z + Sex	+ Trial.z	+ (1+Trial.z||AnimalID),	family = beta_family, data = Both.LookingTP)

Full-null model:

|  | npar | AIC | BIC | logLik | Chisq | Df | Pr(>Chisq) |
| --- | --- | --- | --- | --- | --- | --- | --- |
| null.LookTP | 7 | -1951.7 | -1923.3 | 982.87 |  |  |  |
| red.Both.LookingTP | 9 | -1956.1 | -1919.5 | 987.05 | 8.35 | 2 | 0.02 |

Summary:

|  | Estimate | Std. Error | z-value | p-value | upperCI | lowerCI |
| --- | --- | --- | --- | --- | --- | --- |
| (Intercept) | -2.45 | 0.11 | -22.88 | <0.001 | -2.69 | -2.26 |
| SpeciesWolf | -0.31 | 0.11 | -2.86 | 0.00 | -0.52 | -0.10 |
| Condition.yExperimental | 0.01 | 0.10 | 0.11 | 0.91 | -0.18 | 0.20 |
| Age.z | 0.14 | 0.05 | 2.73 | 0.01 | 0.04 | 0.24 |
| SexM | 0.00 | 0.10 | 0.04 | 0.97 | -0.20 | 0.19 |
| Trial.z | -0.07 | 0.05 | -1.50 | 0.13 | -0.17 | 0.02 |

Posthoc:

| contrast | estimate | SE | df | z.ratio | p.value |
| --- | --- | --- | --- | --- | --- |
| Dog - Wolf | 0.31 | 0.12 | Inf | 2.86 | 0.004 |

### 6.3.7 Looking at the targets – Guided phase

Formula: full.Both.LookingG <- glmmTMB(Looking.at.target.G ~	Species	* Condition.y + Age.z + Sex	+ Trial.z	+ (1+Trial.z||AnimalID),	family = beta_family, data = Both.LookingG)

Full-null model:

|  | npar | AIC | BIC | logLik | Chisq | Df | Pr(>Chisq) |
| --- | --- | --- | --- | --- | --- | --- | --- |
| null.Both.LookingG | 7 | -2938.7 | -2911.1 | 1476.3 |  |  |  |
| full.Both.LookingG | 10 | -2934 | -2894.6 | 1477 | 1.33 | 3 | 0.72 |

Summary:

|  | Estimate | Std. Error | z-value | p-value | upperCI | lowerCI |
| --- | --- | --- | --- | --- | --- | --- |
| (Intercept) | 1.95 | 0.13 | 14.70 | <0.001 | 1.69 | 2.20 |
| SpeciesWolf | 0.11 | 0.17 | 0.61 | 0.54 | -0.24 | 0.41 |
| Condition.yExperimental | -0.01 | 0.14 | -0.08 | 0.93 | -0.26 | 0.25 |
| Age.z | 0.06 | 0.06 | 1.04 | 0.30 | -0.06 | 0.17 |
| SexM | 0.05 | 0.12 | 0.42 | 0.68 | -0.18 | 0.29 |
| Trial.z | -0.12 | 0.06 | -2.14 | 0.03 | -0.22 | 0.00 |
| SpeciesWolf: Condition.yExperimental | -0.22 | 0.24 | -0.89 | 0.37 | -0.65 | 0.24 |

### 6.3.8 Number of commands

Formula: full.Both.commands <-	glmer(Command.Smell ~ Species * Condition.y +	Age.z +	Sex +	Trial.z	+ (1+Trial.z||AnimalID),	family = poisson(), data	= Both.commands)

Full-null model:

|  | npar | AIC | BIC | logLik | Chisq | Df | Pr(>Chisq) |
| --- | --- | --- | --- | --- | --- | --- | --- |
| null.Both.commands | 6 | 489.32 | 507.73 | -238.66 |  |  |  |
| full.Both.commands | 9 | 491.17 | 518.79 | -236.59 | 4.15 | 3 | 0.25 |

Summary:

|  | Estimate | Std. Error | z-value | p-value | upperCI | lowerCI |
| --- | --- | --- | --- | --- | --- | --- |
| (Intercept) | 0.10 | 0.21 | 0.49 | 0.63 | -0.24 | 0.48 |
| SpeciesWolf | 0.33 | 0.33 | 0.98 | 0.33 | -0.38 | 0.91 |
| Condition.yExperimental | -0.22 | 0.17 | -1.28 | 0.20 | -0.51 | 0.20 |
| Age.z | -0.10 | 0.13 | -0.73 | 0.46 | -0.30 | 0.19 |
| SexM | 0.14 | 0.25 | 0.55 | 0.58 | -0.38 | 0.62 |
| Trial.z | 0.17 | 0.10 | 1.78 | 0.08 | -0.01 | 0.37 |
| SpeciesWolf: Condition.yExperimental | -0.21 | 0.34 | -0.61 | 0.54 | -1.01 | 0.40 |

### 6.3.9 Latency

Formula: Both.full.lat.wc <- lmer(LatencySmell_log ~ Condition.y*Species + Age.z + Sex +	Trial.z +	(1+Trial.z+Condition.y.Experimental|AnimalID), data = Both.latency)

Full-null model:

|  | npar | AIC | BIC | logLik | Chisq | Df | Pr(>Chisq) |
| --- | --- | --- | --- | --- | --- | --- | --- |
| Both.null.lat | 8 | 434.92 | 459.52 | -209.46 |  |  |  |
| Both.full.lat | 11 | 435.09 | 468.92 | -206.54 | 5.83 | 3 | 0.12 |

Summary:

|  | Estimate | Std. Error | z-value | upperCI | lowerCI |
| --- | --- | --- | --- | --- | --- |
| (Intercept) | 2.24 | 0.15 | 14.93 | 1.96 | 2.52 |
| Condition.yExperimental | -0.22 | 0.16 | -1.42 | -0.54 | 0.08 |
| SpeciesWolf | 0.38 | 0.20 | 1.94 | -0.01 | 0.77 |
| Age.z | -0.07 | 0.09 | -0.80 | -0.24 | 0.10 |
| SexM | 0.29 | 0.17 | 1.69 | -0.03 | 0.62 |
| Trial.z | 0.17 | 0.10 | 1.65 | -0.04 | 0.37 |
| Condition.yExperimental:SpeciesWolf | 2.24 | 0.15 | 14.93 | 1.96 | 2.52 |

## 6.4 Non-target directed: Interaction Species * Condition

### 6.4.1 Looking at experimenter

Formula: red.Both.LookEPG <- glmmTMB(Look.at.experimenter_sum ~ Condition.y + Species + Age.z	+ Sex +	Trial.z + (1+Trial.z||AnimalID), family=beta_family(), data = Both.LookEPG)

Full-null model:

|  | npar | AIC | BIC | logLik | Chisq | Df | Pr(>Chisq) |
| --- | --- | --- | --- | --- | --- | --- | --- |
| null.Both.LookEPG | 7 | -191.29 | -167.1 | 102.64 |  |  |  |
| full.Both.LookEPG | 10 | -212 | -177.44 | 116 | 26.71 | 3 | <0.001 |

Summary:

|  | Estimate | Std. Error | z-value | p-value | upperCI | lowerCI |
| --- | --- | --- | --- | --- | --- | --- |
| (Intercept) | -0.58 | 0.12 | -4.70 | <0.001 | -0.84 | -0.34 |
| Condition.yExperimental | 0.22 | 0.12 | 1.86 | 0.06 | -0.02 | 0.45 |
| SpeciesWolf | -1.05 | 0.15 | -6.88 | <0.001 | -1.36 | -0.75 |
| Age.z | 0.07 | 0.07 | 0.98 | 0.33 | -0.07 | 0.21 |
| SexM | -0.15 | 0.15 | -1.06 | 0.29 | -0.44 | 0.12 |
| Trial.z | -0.22 | 0.09 | -2.41 | 0.02 | -0.41 | -0.03 |

Posthoc:

| contrast | estimate | SE | df | z.ratio | p.value |
| --- | --- | --- | --- | --- | --- |
| Control - Experimental | -0.22 | 0.12 | Inf | -1.86 | 0.06 |
| Dog - Wolf | 1.05 | 0.15 | Inf | 6.88 | <.0001 |

### 6.4.2 Proximity to experimenter

Formula: full.Both.ProxEP <- glmmTMB(Proximity.to.experimenter_Sum	~ Condition.y*Species +	Age.z +	Sex + Trial.z + (1+Trial.z+Condition.y.Experimental|AnimalID), family =	beta_family(), data = Both.ProxEP)

Full-null model:

|  | npar | AIC | BIC | logLik | Chisq | Df | Pr(>Chisq) |
| --- | --- | --- | --- | --- | --- | --- | --- |
| null.Both.ProxEP | 11 | -121.06 | -83.052 | 71.53 |  |  |  |
| full.Both.ProxEP | 14 | -117.25 | -68.874 | 72.624 | 2.19 | 3 | 0.53 |

Summary:

|  | Estimate | Std. Error | z-value | p-value | upperCI | lowerCI |
| --- | --- | --- | --- | --- | --- | --- |
| (Intercept) | -0.55 | 0.27 | -2.03 | 0.04 | -1.05 | -0.05 |
| Condition.yExperimental | 0.18 | 0.25 | 0.72 | 0.47 | -0.29 | 0.63 |
| SpeciesWolf | 0.30 | 0.36 | 0.83 | 0.41 | -0.35 | 0.94 |
| Age.z | -0.09 | 0.13 | -0.71 | 0.48 | -0.34 | 0.20 |
| SexM | -0.09 | 0.28 | -0.32 | 0.75 | -0.65 | 0.43 |
| Trial.z | -0.04 | 0.12 | -0.33 | 0.74 | -0.27 | 0.19 |
| Condition.yExperimental: SpeciesWolf | -0.61 | 0.41 | -1.50 | 0.13 | -1.33 | 0.14 |

### 6.4.3 High tail

Formula: red.Both.TailHP <- glmmTMB(Tail.wag.high_sum ~ Condition.y + Species + Age.z + Sex +	Trial.z + (1+Trial.z+Condition.y.Experimental|AnimalID),	family = beta_family(), data =	Both.TailHP)

Full-null model:

|  | npar | AIC | BIC | logLik | Chisq | Df | Pr(>Chisq) |
| --- | --- | --- | --- | --- | --- | --- | --- |
| null.Both.TailHP | 11 | -926.64 | -888.63 | 474.32 |  |  |  |
| full.Both.TailHP | 14 | -930.21 | -881.84 | 479.11 | 9.60 | 3 | 0.023 |

Summary:

|  | Estimate | Std. Error | z-value | p-value | upperCI | lowerCI |
| --- | --- | --- | --- | --- | --- | --- |
| (Intercept) | -0.90 | 0.45 | -2.00 | 0.05 | -1.74 | -0.02 |
| Condition.yExperimental | 0.06 | 0.26 | 0.23 | 0.82 | -0.44 | 0.55 |
| SpeciesWolf | -1.96 | 0.57 | -3.47 | 0.00 | -3.11 | -0.80 |
| Age.z | -0.51 | 0.27 | -1.90 | 0.06 | -1.09 | 0.07 |
| SexM | 0.37 | 0.58 | 0.63 | 0.53 | -0.78 | 1.50 |
| Trial.z | -0.06 | 0.08 | -0.77 | 0.44 | -0.22 | 0.09 |

Posthoc:

| contrast | estimate | SE | df | z.ratio | p.value |
| --- | --- | --- | --- | --- | --- |
| Dog - Wolf | 1.96 | 0.56 | Inf | 3.46 | <0.001 |

### 6.4.4 Low tail

Formula: red.Both.TailLP <- glmmTMB(Tail.wag.low_sum ~ Condition.y+Species	+ Age.z	+ Sex +	Trial.z	+ (1+Trial.z+Condition.y.Experimental|AnimalID), family	= beta_family(),	data =	Both.TailLP)

Full-null model:

|  | npar | AIC | BIC | logLik | Chisq | Df | Pr(>Chisq) |
| --- | --- | --- | --- | --- | --- | --- | --- |
| null.Both.TailLP | 11 | -804.18 | -766.17 | 413.09 |  |  |  |
| full.Both.TailLP | 14 | -805.99 | -757.62 | 417 | 7.81 | 3 | 0.05 |

Summary:

|  | Estimate | Std. Error | z-value | p-value | upperCI | lowerCI |
| --- | --- | --- | --- | --- | --- | --- |
| (Intercept) | 0.31 | 0.39 | 0.80 | 0.42 | -0.51 | 0.88 |
| Condition.yExperimental | -0.04 | 0.17 | -0.21 | 0.83 | -0.34 | 0.32 |
| SpeciesWolf | 1.52 | 0.51 | 3.00 | 0.00 | 0.49 | 2.00 |
| Age.z | 0.38 | 0.20 | 1.86 | 0.06 | -0.05 | 0.67 |
| SexM | -0.23 | 0.40 | -0.58 | 0.56 | -1.03 | 0.57 |
| Trial.z | -0.30 | 0.16 | -1.85 | 0.06 | -0.67 | -0.11 |

Posthoc:

| contrast | estimate | SE | df | z.ratio | p.value |
| --- | --- | --- | --- | --- | --- |
| Dog - Wolf | -1.52 | 0.51 | Inf | -3 | <0.003 |

### 6.4.5 Not accomplished

Formula: red.Both.NotAcc <- glmer(cbind(Not.accomplished, TotalTrials-Not.accomplished) ~	Condition.y + Species +	Age.z +	Sex + (1|AnimalID), family=binomial, data=Both.NotAcc)

Full-null model:

|  | npar | AIC | BIC | logLik | Chisq | Df | Pr(>Chisq) |
| --- | --- | --- | --- | --- | --- | --- | --- |
| null.Both.NotAcc | 4 | 141.36 | 148.02 | -66.682 |  |  |  |
| full.Both.NotAcc | 7 | 133.77 | 145.42 | -59.887 | 13.59 | 3 | 0.004 |

Summary:

|  | Estimate | Std. Error | z-value | p-value | upperCI | lowerCI |
| --- | --- | --- | --- | --- | --- | --- |
| (Intercept) | -1.73 | 0.51 | -3.41 | 0.00 | -2.59 | -0.97 |
| Condition.yF | -0.65 | 0.37 | -1.77 | 0.08 | -1.51 | 0.27 |
| SpeciesWolf | 2.11 | 0.64 | 3.29 | 0.00 | 0.68 | 3.19 |
| Age.z | -0.80 | 0.32 | -2.46 | 0.01 | -1.40 | -0.23 |
| SexM | -0.09 | 0.57 | -0.15 | 0.88 | -1.09 | 1.04 |

Posthoc:

| contrast | estimate | SE | df | z.ratio | p.value |
| --- | --- | --- | --- | --- | --- |
| Dog - Wolf | -2.11 | 0.64 | Inf | -3.29 | 0.001 |
| Control - Experimental | 0.65 | 0.36 | Inf | 1.77 | 0.07 |

### 6.4.6 Total trials

Formula: full.Both.TotalTrials=glmer(TotalTrials	~ Condition.y*Species +	Age.z +	Sex +	(1|AnimalID), family=poisson, data=Both.TotalTrials)

Full-null model:

|  | npar | AIC | BIC | logLik | Chisq | Df | Pr(>Chisq) |
| --- | --- | --- | --- | --- | --- | --- | --- |
| null.Both.TotalTrials | 4 | 154.89 | 161.54 | -73.443 |  |  |  |
| full.Both.TotalTrials | 7 | 158.76 | 170.41 | -72.38 | 2.13 | 3 | 0.55 |

Summary:

|  | Estimate | Std. Error | z-value | p-value | upperCI | lowerCI |
| --- | --- | --- | --- | --- | --- | --- |
| (Intercept) | 1.80 | 0.13 | 13.65 | <0.001 | 1.57 | 2.04 |
| Condition.yF | -0.03 | 0.17 | -0.17 | 0.87 | -0.33 | 0.27 |
| SpeciesWolf | -0.25 | 0.21 | -1.18 | 0.24 | -0.70 | 0.13 |
| Age.z | 0.03 | 0.07 | 0.45 | 0.66 | -0.09 | 0.15 |
| SexM | -0.01 | 0.14 | -0.09 | 0.93 | -0.21 | 0.29 |
| Condition.yF:SpeciesWolf | 0.08 | 0.29 | 0.28 | 0.78 | -0.49 | 0.66 |

### 6.4.7 Termination

Formula: full.Both.Termination=glmer(Termination ~ Condition.y*Species + Age.z + Sex +	(1|AnimalID), family=binomial, data=Both.Termination)

Full-null model:

|  | npar | AIC | BIC | logLik | Chisq | Df | Pr(>Chisq) |
| --- | --- | --- | --- | --- | --- | --- | --- |
| null.Both.Termination | 4 | 34.62 | 41.27 | -13.31 |  |  |  |
| full.Both.Termination | 7 | 29.44 | 41.08 | -7.72 | 11.18 | 3 | 0.01 |

Summary:

|  | Estimate | Std. Error | z-value | p-value | upperCI | lowerCI |
| --- | --- | --- | --- | --- | --- | --- |
| (Intercept) | -2.38E+04 | 6.65E+02 | -3.58E+01 | <2e-16 | -23814.68 | -23760.61 |
| Condition.yF | 2.38E+04 | 6.65E+02 | 3.58E+01 | <2e-16 | 23746.74 | 23808.67 |
| SpeciesWolf | 2.38E+04 | 6.65E+02 | 3.58E+01 | <2e-16 | 23752.93 | 23812.54 |
| Age.z | -3.87E-01 | 2.39E+00 | -1.62E-01 | 0.87 | -15.17 | 14.70 |
| SexM | -1.55E-01 | 5.39E+00 | -2.90E-02 | 0.97 | -29.81 | 23.58 |
| Condition.yF:SpeciesWolf | -2.38E+04 | 6.65E+02 | -3.58E+01 | <2e-16 | -23808.79 | -23741.957 |

Posthoc:

| contrast | estimate | SE | df | z.ratio | p.value |
| --- | --- | --- | --- | --- | --- |
| CDog-FDog | -2.38E+04 | 665 | Inf | -35.79 | <.0001 |
| CDog-CWolf | -2.38E+04 | 665 | Inf | -35.79 | <.0001 |
| CDog-FWolf | -2.38E+04 | 665 | Inf | -35.79 | <.0001 |
| FDog-CWolf | -0.76 | 5.84 | Inf | -0.13 | 0.99 |
| FDog-FWolf | -0.68 | 5.76 | Inf | -0.11 | 0.99 |
| CWolf-FWolf | 0.08 | 4.57 | Inf | 0.01 | 1 |

### 6.4.8 Displacement – Test phase

Formula: full.Both.DisplaceTment=glmer(Displacements_sum ~ Condition.y*Species + Age.z + Sex	+ offset(log(Trial.duration.T)) + (1+Trial.z+Condition.y.Experimental||AnimalID),	family=poisson,	data=Both.DisplaceT)

Full-null model:

|  | npar | AIC | BIC | logLik | Chisq | Df | Pr(>Chisq) |
| --- | --- | --- | --- | --- | --- | --- | --- |
| null.Both.DisplaceTment | 6 | 402.52 | 422.74 | -195.26 |  |  |  |
| full.Both.DisplaceTment | 9 | 405.55 | 435.89 | -193.78 | 2.97 | 3 | 0.40 |

Summary:

|  | Estimate | Std. Error | z-value | p-value | upperCI | lowerCI |
| --- | --- | --- | --- | --- | --- | --- |
| (Intercept) | -4.59 | 0.31 | -14.92 | <0.001 | -5.31 | -4.04 |
| Condition.yExperimental | 0.37 | 0.33 | 1.12 | 0.26 | -0.22 | 0.94 |
| SpeciesWolf | 0.54 | 0.39 | 1.38 | 0.17 | -0.21 | 1.30 |
| Age.z | 0.01 | 0.15 | 0.06 | 0.95 | -0.36 | 0.27 |
| SexM | 0.18 | 0.30 | 0.58 | 0.56 | -0.31 | 0.69 |
| Condition.yExperimental: SpeciesWolf | -0.29 | 0.46 | -0.63 | 0.53 | -1.08 | 0.71 |

### 6.4.9 Nose licks – Guided phase

Formula: full.Both.Noselick.Gment=glmer(Displacements_sum ~	Condition.y*Species + Age.z + Sex	+ Trial.z	+ offset(log(Trial.duration.G)) + (1+Trial.z + Condition.y.Experimental||AnimalID),	family=poisson,	data=Both.Noselick.G)

Full-null model:

|  | npar | AIC | BIC | logLik | Chisq | Df | Pr(>Chisq) |
| --- | --- | --- | --- | --- | --- | --- | --- |
| null.Both.Noselick.Gment | 6 | 464.99 | 485.21 | -226.49 |  |  |  |
| full.Both.Noselick.Gment | 10 | 471.53 | 505.23 | -225.76 | 1.46 | 4 | 0.83 |

Summary:

|  | Estimate | Std. Error | z-value | p-value | upperCI | lowerCI |
| --- | --- | --- | --- | --- | --- | --- |
| (Intercept) | -3.79 | 0.32 | -11.81 | 0.001 | -4.14 | -2.77 |
| Condition.yExperimental | -0.34 | 0.30 | -1.15 | 0.25 | -1.69 | -0.05 |
| SpeciesWolf | -0.04 | 0.44 | -0.09 | 0.93 | -0.67 | 1.75 |
| Age.z | 0.01 | 0.18 | 0.03 | 0.98 | -0.47 | 0.36 |
| SexM | 0.30 | 0.36 | 0.82 | 0.41 | -0.71 | 1.41 |
| Trial.z | 0.01 | 0.11 | 0.06 | 0.96 | -0.40 | 0.30 |
| Condition.yExperimental:  SpeciesWolf | 0.40 | 0.45 | 0.87 | 0.38 | -0.94 | 1.69 |

## 6.5 Wilcoxon Signed-Rank Tests: CF-WSC Dogs (Control vs. Experimental condition)

### 6.5.1 Proximity to target – Guided phase

W=27, p=0.038

### 6.5.2 Proximity to target – Test phase

W=26, p=0.34

### 6.5.3 Sniffing target

W=28, p=0.42

### 6.5.4 Engaging with target

W=33, p=0.68

### 6.5.5 Looking at target – Guided phase

W=23, p=0.23

### 6.5.6 Command accomplished

W=20, p=0.15

### 6.5.7 Latency to accomplish command

W=29, p=0.47

### 6.5.8 Number of commands

W=20, p=0.15

## 6.6 Wilcoxon Signed-Rank Tests: CF-WSC Wolves (Control vs. Experimental condition)

### 6.6.1 Proximity to target – Guided phase

W=7, p=0.30

### 6.6.2 Proximity to target – Test phase

W=7, p=0.53

### 6.6.3 Sniffing target

W=7, p=0.30

### 6.6.4 Engaging with target

W=1, p=0.11

### 6.6.5 Looking at target – Guided phase

W=16, p=0.81

### 6.6.6 Looking at target – Test phase

W=3, p=1

### 6.6.7 Command accomplished

W=3, p=0.63

### 6.6.8 Latency to accomplish command

W=4, p=0.86

### 6.6.9 Number of commands

W=3, p=0.63

## 6.7 Mann-Whitney U Test: CF-WSC Dogs Experimental vs. Pet dogs Experimental condition

Pet dog data was taken from Capitain et al. 2025^[1]^

### 6.7.1 Proximity to target – Test phase

W=379, p=0.21

### 6.7.2 Sniffing target

W=235, p=0.83

### 6.7.3 Engaging with target

W=0, p=0.14

### 6.7.4 Looking at target – Guided phase

W=441, p=0.57

### 6.7.5 Looking at target – Test phase

W=468, p=0.79

1. Capitain, S., Range, F., & Marshall-Pescini, S. (2025). Not just avoidance: Dogs show subtle individual differences in reacting to human fear chemosignals. *Frontiers in Veterinary Science*, *12*, 1679991.
